# Polarised Clathrin-Mediated Endocytosis of EGFR During Chemotactic Invasion

**DOI:** 10.1111/tra.12165

**Published:** 2014-03-20

**Authors:** Laura Jane Mutch, Jake Davey Howden, Emma Poppy Louise Jenner, Natalie Sarah Poulter, Joshua Zachary Rappoport

**Affiliations:** 1 School of Biosciences, The University of BirminghamEdgbaston, Birmingham, B15 2TT, UK; 2 Centre for Cardiovascular Research, Institute for Biomedical Research, The College of Medical and Dental Sciences, The University of BirminghamEdgbaston, Birmingham, B15 2TT, UK

**Keywords:** breast cancer, chemotactic invasion, EGFR, endocytosis, MDA-MB-231

## Abstract

Directed cell migration is critical for numerous physiological processes including development and wound healing. However chemotaxis is also exploited during cancer progression. Recent reports have suggested links between vesicle trafficking pathways and directed cell migration. Very little is known about the potential roles of endocytosis pathways during metastasis. Therefore we performed a series of studies employing a previously characterised model for chemotactic invasion of cancer cells to assess specific hypotheses potentially linking endocytosis to directed cell migration. Our results demonstrate that clathrin-mediated endocytosis is indispensable for epidermal growth factor (EGF) directed chemotactic invasion of MDA-MB-231 cells. Conversely, caveolar endocytosis is not required in this mode of migration. We further found that chemoattractant receptor (EGFR) trafficking occurs by clathrin-mediated endocytosis and is polarised towards the front of migrating cells. However, we found no role for clathrin-mediated endocytosis in focal adhesion disassembly in this migration model. Thus, this study has characterised the role of endocytosis during chemotactic invasion and has identified functions mechanistically linking clathrin-mediated endocytosis to directed cell motility.

## Introduction

Directed cell migration underlies many physiological processes. Under most circumstances, including embryonic development, wound repair and immune responses, cell migration is beneficial to the normal growth and survival of an organism. However, cell migration can also promote progression of cancer through angiogenesis and metastasis and, under these circumstances, has a negative impact on the survival of the organism. Cancer metastasis is strongly associated with a poor prognosis and chance of survival; as such the prevention of metastasis is a key target for therapeutic intervention (1).

During the intravasation and extravasation stages of metastasis, cells migrate towards a source of chemoattractant in a process called chemotaxis (2). This process is triggered by the binding of chemoattractant molecules to cell surface receptors. Epidermal growth factor (EGF) binding to its receptor (EGFR) has been identified as a potent chemoattractant stimulus for metastatic cancer cells (3). Activation of chemoattractant receptors results in a complex signalling cascade that leads to polarisation of the cell in the direction of migration, increased contractility and subsequent directed motility (4).

In order to control the directionality and magnitude of migration, the availability of chemoattractant receptors and cell adhesion molecules on the cell surface must be carefully regulated. Endocytosis is the first step of endocytic recycling and, as such, is important for regulation of receptor signalling, and has also been shown to be important for migration in a number of different systems (5,6). Recently, it has been demonstrated that inhibiting endocytosis had a negative effect on Madin-Darby canine kidney (MDCK) epithelial cell migration in a wound healing model (7). Similarly, endocytosis has been shown to be important for platelet-derived growth factor-dependent chemotaxis of fibroblasts in a Boyden chamber (8).

Polarisation of vesicle trafficking pathways, such as endocytosis, along the migratory axis has been suggested as important for the promotion of cell migration (9–11). This area is widely debated due to uncertainty in the precise vesicle trafficking pathways involved and the specific membrane domains to which these might be polarised. It has recently been suggested that during epithelial wound healing clathrin-mediated endocytosis takes place in the middle-to-front area of the cell (12). Conversely, in this same system caveolar endocytosis is polarised towards the rear of the cell (6,7). This polarisation, however, may vary in different cell types and experimental systems.

To date there has been limited research into the role of endocytosis during chemotactic invasion. Potential endocytic cargo which may have a role in migration include focal adhesion components and chemoattractant receptors. Focal adhesions are involved in connecting cells to the extra-cellular matrix and providing the traction force allowing cells to move (13–15). Thus, factors affecting the disassembly of focal adhesions could have roles in controlling the rate and magnitude of migration, and endocytosis of integrin cell adhesion molecules from disassembling focal adhesions has been proposed to occur in certain situations (10,11,16,17).

Chemoattractant receptors are utilised to sense the surrounding gradient of chemoattractant and enable directionality of cell migration. The EGFR (also known as ErbB1 and Her1) is linked to many vital cellular processes such as cell survival, proliferation, differentiation and migration (18), and deregulation of EGFR can be oncogenic. EGFRs are frequently overexpressed in breast cancer and this overexpression appears to correlate with increased aggressiveness of the cancer (19).

There is conflicting evidence over which route(s) of internalisation EGFR employ(s) and what its fate is following endocytosis. Some groups describe a clathrin-dependent route for internalisation while others provide evidence that the route can be clathrin-independent (20–23). Here we aim to specifically elucidate the route of internalisation of the EGFR during chemotactic invasion of the highly migratory breast cancer cell line MDA-MB-231 and test specific hypotheses mechanistically linking endocytosis to chemotactic invasion. Our results demonstrate that dynamin-dependent clathrin-mediated endocytosis is essential for EGF-directed migration. Alternatively we found caveolar endocytosis to be entirely dispensable. While we find no role for clathrin-mediated endocytosis in focal adhesion disassembly during chemotactic invasion, we have demonstrated that clathrin-mediated endocytosis is polarised towards the front of migrating cells and that EGFR internalises via this route. In summary, we propose a model where clathrin-mediated endocytosis of EGFR is polarised towards the front of migrating cells and this polarised trafficking is necessary for migration in an EGF-containing environment. Additionally we suggest that clathrin-mediated endocytosis does not play a role in focal adhesion disassembly in this migratory model.

## Results

### The effect of dynamin inhibition on chemotactic invasion

In order to investigate potential mechanistic roles for endocytosis during chemotactic invasion we began by assessing the effect of inhibiting different endocytic pathways during EGF-directed migration in an assay system we recently developed (24). Briefly, our agarose spot assay for chemotactic invasion suspends chemoattractants (e.g. EGF) in a small drop of low melting point agarose adhered to a coverslip. Cells subsequently plated into wells containing agarose spots exhibit directed chemotactic invasion underneath the agarose spots, and can be observed through a wide variety of microscopy techniques.

**Figure 1:**
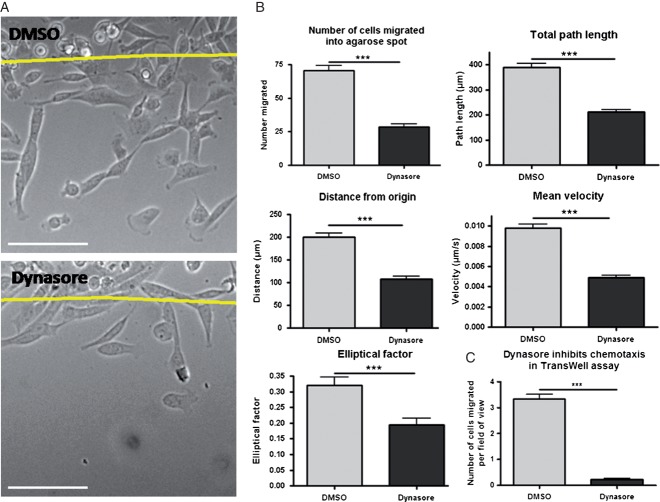
Dynasore inhibits MDA-MB-231 chemotactic invasion into EGF spots. A) Representative images treated with DMSO or Dynasore after 14 h migration into an agarose spot containing EGF. Line denotes edge of the agarose spot. Scale bars 100 µm. B) Results of tracking cells over the course of the timelapses. Parameters analysed include total number of cells migrated, total path length, distance from origin, average velocity and elliptical factor (found by dividing width of cell by length to give a number between 0 and 1). *n* = 36 fields of view per treatment for ‘number of cell migrated’ graph, *n* = 100 cells per treatment for all other analyses. C) Dynasore inhibits chemotaxis towards EGF in Transwells. Number of cells migrated per field of view following DMSO and Dynasore treatment. *n* ≥ 50 fields of view per treatment.

The GTPase dynamin is known to be involved in both clathrin-mediated endocytosis and caveolar endocytosis, as well as having possible roles in other less characterised endocytosis pathways (5,25,26). Therefore, we utilised Dynasore, a small module inhibitor of dynamin, to determine if dynamin-dependent endocytosis is required for chemotactic invasion (27). In preliminary control experiments using fluorescent transferrin as a marker for clathrin-mediated endocytosis and fluorescent cholera toxin B (CTxB) as a marker for caveolar endocytosis we confirmed that Dynasore inhibits both these routes of internalisation (Figure S1, Supporting Information). When we treated MDA-MB-231 cells in the chemotactic invasion assay with Dynasore we saw significant differences in a number of parameters investigated, including an overall reduction in the total number of cells which migrated (60% decrease, Figure 1A and B). Investigating the 40% of cells which still migrated led us to find perturbations in the total path length travelled (45% decrease), the distance travelled from their start point (46% decrease) and their average velocity (50% decrease, Figure 1B). We next chose to validate these results with conventional Transwell-based experiments. As depicted in Figure 1C, Dynasore treatment resulted in a nearly complete inhibition of EGF-directed motility in these studies. Thus, dynamin-dependent endocytosis plays a major role in EGF-directed chemotactic invasion in MDA-MB-231 cells.

**Figure 2:**
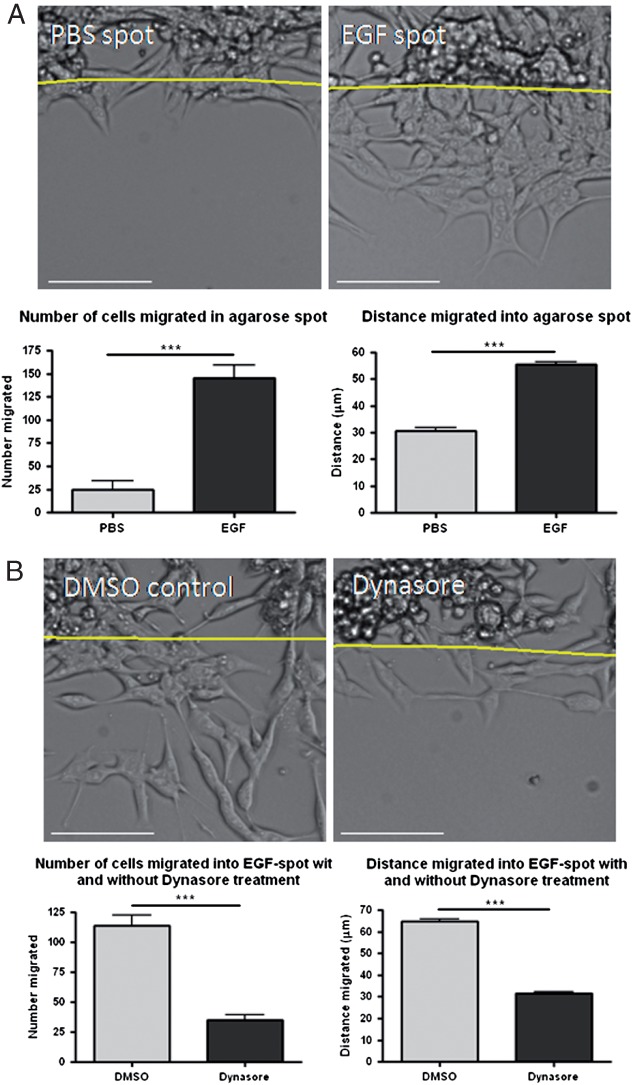
Dynasore inhibits PDACs chemotactic invasion into EGF spots. A) The total number of PDACs cells migrated into the spot and the distance migrated by cells was significantly higher in EGF spots compared to control PBS spots. Line denotes edge of the agarose spot. B) Dynasore treatment caused a decrease in the number of PDACs cells migrated and distance migrated into EGF spots. Scale bars are 100 µm. *n* ≥ 12 fields of view per treatment.

In order to further expand the applicability of our studies we analysed a second cell line. Pancreatic ductal adenocarcinoma (PDAC) cells derived from a pancreatic tumour, have been previously characterised and are chemotactic and metastatic (28–30). As depicted in Figure 2A, as with MDA-MB-231 cells, PDACs demonstrate chemotactic invasion into agarose spots containing EGF. Both the number of migratory cells, and distance travelled were significantly greater in EGF spots compared to control spots containing PBS in place of chemoattractant. Furthermore, as with MDA-MB-231 cells, chemotactic invasion of PDACs was sensitive to Dynasore treatment (Figure 2B). Importantly these results are similar to those obtained following Dynasore treatment in PDACs cells in studies employing other cell migration models, including EGF stimulated wound healing (30), and suggest that dynamin function is required for chemotactic invasion in multiple cell platforms.

**Figure 3:**
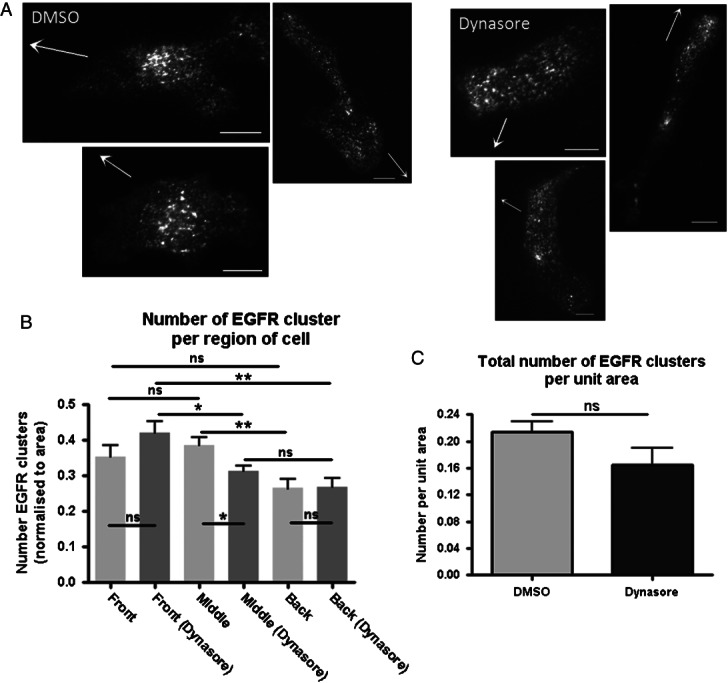
Dynasore causes redistribution of EGFR during chemotactic invasion. A) Representative images of cells used for EGFR cluster number counts in migrating cells treated with DMSO and Dynasore. Scale bars 10 µm. B) Quantification of EGFR clusters by cell region with and without Dynasore treatment. C) Overall there is no significant increase in the number of clusters following Dynasore treatment. *n* = 12 cells per treatment.

Previously we have shown that activated growth factor receptors cluster to sites of endocytosis (21,31). As depicted in Figure 3 when we visualised GFP-tagged EGFR by total internal reflection fluorescence (TIRF) microscopy performed on cells undergoing EGF-directed chemotactic invasion we observed EGFR clusters in the front, middle and back of the adherent plasma membrane (32,33). Importantly, in non-stimulated cells not exposed to EGF this construct does not form clusters in the plasma membrane of MDA-MB-231 cells (Figure S2). Thus, we next set out to determine if Dynasore treatment altered the distribution of EGFR clusters.

As depicted in Figure 3, when comparing the distribution of EGFR clusters in the adherent plasma membrane of cells migrating in EGF spots Dynasore treatment caused a decrease in the proportion of clusters in the middle of the cell, and an increase in those towards the front, although only the former was statistically significant. In further analyses we found that following Dynasore treatment, but not in untreated control cells, there were more EGFR clusters in the front of the cell relative to the middle and back of the adherent plasma membrane. Thus, Dynasore treatment causes a redistribution of EGFR clusters, potentially suggesting that endocytosis of activated EGFR is polarised during EGF-directed chemotactic invasion. Furthermore, consistent with our previous work investigating the endocytic trafficking of activated EGFR in non-motile cells, Dynasore treatment did not increase the total number of EGFR clusters in the adherent plasma membrane (Figure 3C); previously we demonstrated that inhibition of clathrin-mediated endocytosis with siRNA silencing of α-adaptin similarly did not increase the number of EGFR clusters in EGF stimulated cells (21).

One potential role previously suggested for endocytosis in cell migration is internalisation of integrins from disassembling focal adhesions, although this phenomenon has not been universally observed (7,34–36). Thus we investigated whether Dynasore treatment causes a similar alteration in the distribution of focal adhesions in migrating MDA-MB-231 cells.

**Figure 4:**
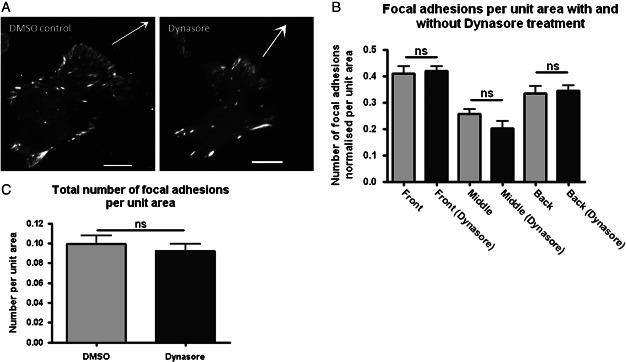
Dynasore does not cause redistribution of focal adhesions during chemotactic invasion. A) Representative images of paxillin-mRFP in cells migrating towards EGF. Scale bars are 10 µm. B) Quantification of number of focal adhesions in the front, middle and back regions of migrating cells. C) Overall there is no significant increase in the number of focal adhesions following Dynasore treatment. *n* = 25 cells per treatment.

We chose to employ paxillin-mRFP for these studies, which should be present in all focal adhesions (37). As depicted in Figure 4, Dynasore treatment did not cause a redistribution of focal adhesions, within each cell region (front, middle and back), the number of focal adhesions was similar with and without Dynasore treatment. Additionally, Dynasore treatment did not increase the total number of focal adhesions per cell (Figure 4C). Thus, in contrast with EGFR, focal adhesion distribution does not seem, in this model, to depend on dynamin function.

### Clathrin-mediated endocytosis of EGFR during chemotactic invasion

**Figure 5:**
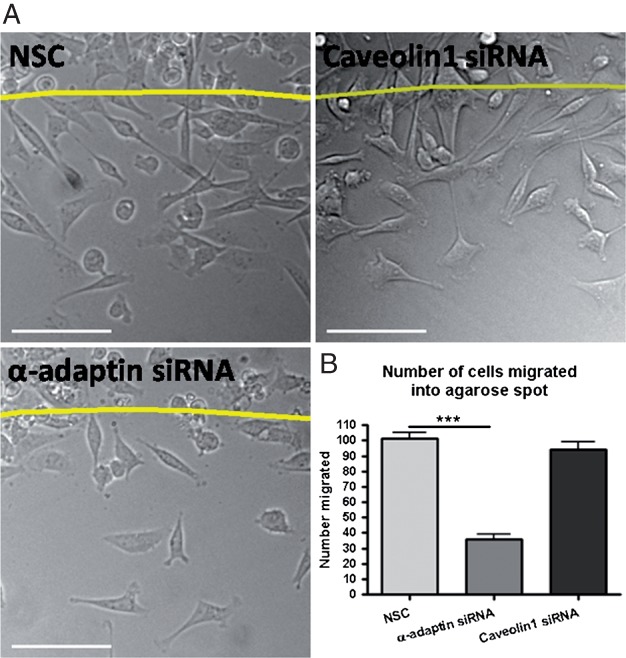
siRNA silencing of α-adaptin inhibits MDA-MB-231 migration into EGF spots. A) Representative images treated with control siRNA, α-adaptin siRNA and caveolin1 siRNA after 14 h migration into an agarose spot containing EGF. Scale bars are 100 µm. B) Quantification of total number of cells migrated under siRNA conditions. Line denotes edge of the agarose spot. *n* = 22 fields of view per treatment. NSC = non-silencing control siRNA.

Dynamin functions in multiple endocytosis pathways, as well as in other cellular processes relevant to cell migration (5,25,26,38). In order to specifically determine the endocytosis pathway(s) that might be important for EGF-directed migration we performed a series of RNAi studies. We employed previously validated siRNA sequences to achieve silencing of α-adaptin, a component of the AP2 complex necessary for clathrin-medicated endocytosis, and caveolin1, necessary for caveolar endocytosis (31). Knock-down was confirmed by western blot (Figure S3A), and inhibition of clathrin-mediated endocytosis and caveolar endocytosis were validated by inhibition of transferrin and CTxB uptake, respectively (Figure S3B). Using siRNA against α-adaptin we saw a large decrease in the total number of cells able to migrate into the agarose spot (65% decrease), conversely, knock-down of caveolin1 had no effect on cell migration (Figure 5A and B). Thus these results indicate that there is a requirement for clathrin-mediated endocytosis in the process of EGF-directed chemotactic invasion, but that caveolar endocytosis is dispensable.

**Figure 6:**
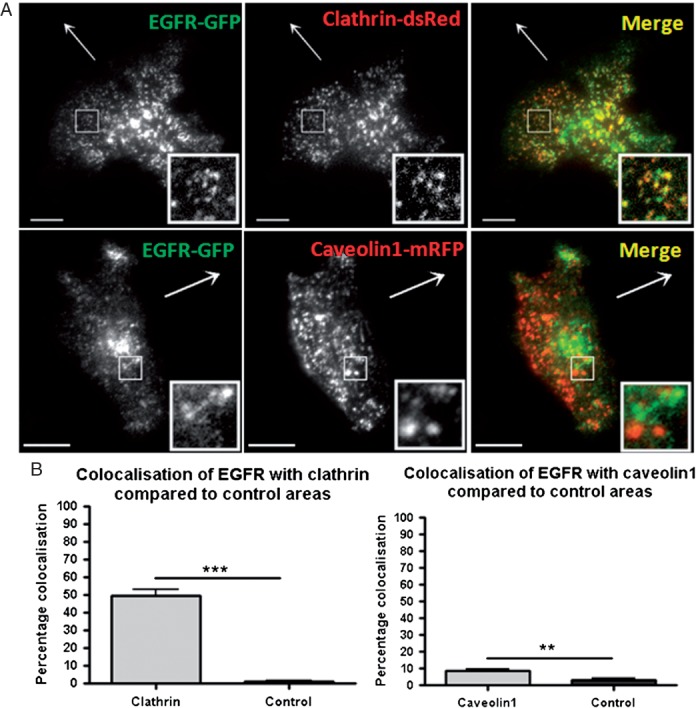
Colocalisation between clathrin-dsRed and EGFR-GFP. A) Representative images of EGFR-GFP with clathrin-dsRed or caveolin1-mRFP in cells migrating towards EGF. Scale bars 10 µm. B) Quantification of colocalisation of EGFR-GFP with clathrin-dsRed and caveolin1-mRFP. *n* = 14 cells for each experiment.

Becausewe had observed a necessity for clathrin-mediated endocytosis in EGF-directed migration we predicted that this pathway might be the route of internalisation of EGFR following ligand binding in this model of migration. Thus, in the agarose spot assay migrating cells expressing GFP-tagged EGFR and clathrin-dsRed were imaged by TIRF microscopy and the degree of colocalisation between the two was quantified. As depicted in Figure 6, significant colocalisation between EGFR and clathrin was observed when compared to ‘control’ regions devoid of EGFR spots. As clathrin-independent endocytosis, such as caveolar endocytosis, has been suggested to play a role in EGFR internalisation in certain situations (20–23) we performed similar analyses assessing the degree of colocalisation between EGFR-GFP and caveolin1-mRFP. In stark contrast to the colocalisation observed between EGFR and clathrin, only approximately 5% of EGFR clusters colocalised with caveolin1, almost 10-fold less than the colocalisation seen with clathrin (Figure 6A and B).

**Figure 7:**
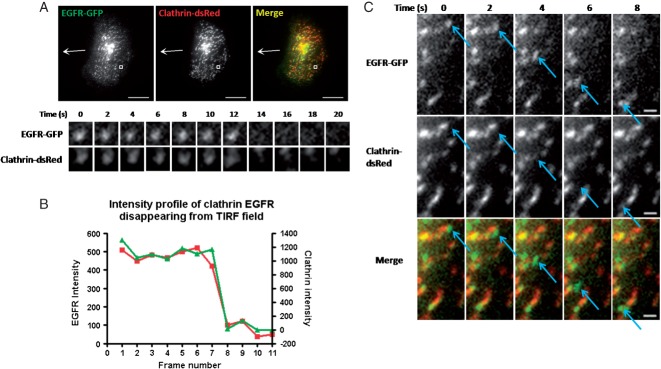
Live-cell TIRF imaging of clathrin-dsRed and EGFR-GFP. A) Representative image to show an instance of both markers leaving the TIRF field at the same time. Scale bars are 10 µm. B) Quantification of the disappearance event depicted in (A). C) Directed motility of an EGFR cluster devoid of clathrin. Scale bars are 1 µm.

Clathrin and EGFR colocalisation during endocytosis was also validated by live cell TIRF imaging and instances of both markers simultaneously disappearing from the TIRF field were observed (Figure 7A and B). However, in these live-cell studies dynamic EGFR spots devoid of clathrin were also tracked. As depicted in Figure 7C, laterally motile EGFR spots, potentially uncoated endocytic structures or post-Golgi carriers, were observed. Thus, these data suggest that during EGF-directed chemotactic invasion activated EGFR enters the cell via clathrin-mediated endocytosis.

**Figure 8:**
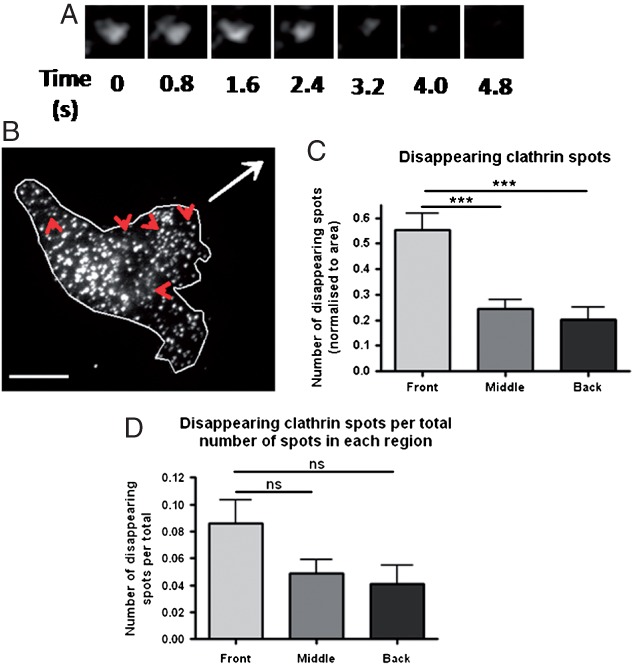
Clathrin spot disappearance is polarised to the front of migrating cells. A) Representative images of a clathrin spot disappearing from the TIRF imaging field. B) Representative image of a clathrin-dsRed expressing migrating cell with instances of clathrin disappearance circled. Scale bars are 10 µm. C) Quantification of clathrin disappearance by cell region. D) Quantification of clathrin disappearance compared to total number of spots in each region. *n* = 18 cells.

Dynasore treatment caused a redistribution of EGFR spots in migrating cells (Figure 3), suggesting the possibility of polarised endocytosis in migrating cells. In order to further investigate this possibility we imaged clathrin-dsRed by live-cell TIRF microscopy in cells migrating in the agarose spot assay. We looked for instances where clathrin disappeared from the evanescent field to indicate an internalisation event. The disappearance of clathrin from the evanescent field was taken to be due to endocytosis only if it was not due to photobleaching, if it occurred rapidly (within 10 frames of imaging) and if it did not reappear in the evanescent field within 10 frames of its disappearance. It was found that over the course of these timelapses significantly more clathrin internalisation events occurred at the front of migrating cells, as opposed to the middle or the back (Figure 8A–C). Furthermore, although it seemed that clathrin-coated pits at the front of the cell are more likely to undergo endocytosis, this effect was not statistically significant (Figure 8D). Thus, taken together with the above results, during EGF-directed migration EGFR is internalised by clathrin-mediated endocytosis and that this internalisation is polarised towards the front of migrating cells.

**Figure 9:**
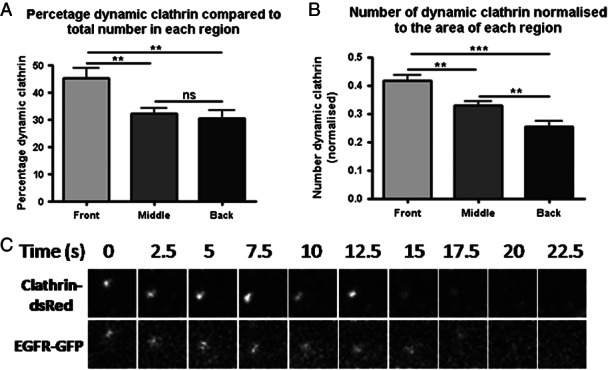
Active clathrin in migrating PDAC cells. A) Graph to show that there is a higher proportion of active or internalising clathrin compared to total clathrin at the front of migrating PDAC cells compared to the middle or the back. B) Graph to show that there is more active or internalising clathrin at the front of migrating PDAC cells when normalised to the area of each region. *n* = 12 cells. C) An instance to show clathrin-dsRed and EGFR-GFP internalising from the TIRF field together.

We also tested whether these observations could be recapitulated in PDAC cells. Live-cell TIRF imaging of clathrin in migrating PDAC cells revealed a very strong preponderance of highly dynamic clathrin. As described in our previous work, when imaged by TIRF microscopy, clathrin can either be static, laterally mobile or disappear vertically into the cell (12,39). Furthermore, it has previously been suggested that movement of clathrin along microtubules can occur prior or subsequent to the endocytosis event (35,39). Thus, we quantified the ‘dynamic’ population of clathrin in migrating PDAC cells, combining those that disappeared vertically as well as those that moved laterally in the plane of the plasma membrane. As it has previously been demonstrated that individual microtubule tracks near the adherent cell surface can curve axially, potentially out of the TIRF field (40), we followed each clathrin spot present in the first image of a 1 min TIRF video. As previously described, we divided the cell into front, middle and back regions. Within each region we counted the number of dynamic clathrin spots. Similar to our results obtained in MDA-MB-231 cells, there were significant differences between the clathrin in the front of the cell, relative to the middle or back. When analysed as a fraction of the total number of spots per region, the front was significantly more dynamic (Figure 9A). Similarly, when we normalised to the area of each region, we found the front contained more dynamic clathrin than the middle or back (Figure 9B). Additionally, we found that EGFR and clathrin colocalised in PDACs, including within spots disappearing from the cell surface (Figure 9C). Thus, we have shown in both MDA-MB-231 and PDAC cells that (1) EGF-directed chemotaxis is dynamin dependant, (2) clathrin dynamics are polarised to the front of the migrating cell, and (3) that clathrin and EGFR can colocalise including during internalisation.

### Clathrin-mediated endocytosis is not involved in focal adhesion disassembly

The inability of Dynasore treatment to cause redistribution of focal adhesions in migrating cells suggests that endocytosis is not required for focal adhesion disassembly in this model. To further test this hypothesis we investigated whether there was a role for clathrin-mediated endocytosis in focal adhesion disassembly in migrating MDA-MB-231 cells. Thus, we investigated the potential colocalisation between clathrin and a previously validated focal adhesion marker GFP-β3-integrin in cells migrating towards EGF in the agarose spot assay (37,41).

**Figure 10:**
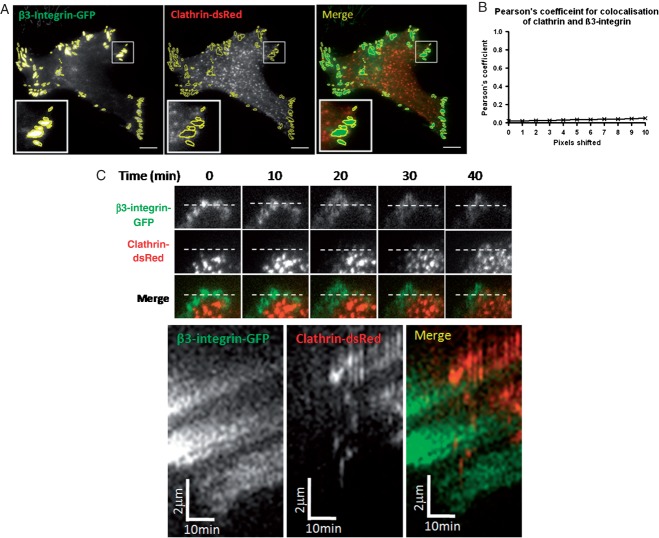
Clathrin-dsRed does not co-localise with focal adhesions using GFP-β3-integrin as the focal adhesion marker. A) Representative images with focal adhesions circled from the GFP-β3-integrin image to assess colocalisation with clathrin. Scale bars are 10 µm. B) Pearson's coefficient of GFP-β3-integrin with clathrin-dsRed. *n* = 30 cells. C) Kymograph to show lack of co-localisation between clathrin-dsRed and GFP-β3-integrin labelled focal adhesions. A 10 µm line was drawn across two disassembling focal adhesions and a kymograph across this region demonstrated no co-localisation of clathrin during focal adhesion disassembly.

As depicted in Figure 10, colocalisation of clathrin and GFP-β3-integrin was not evident. Only approximately 7% of focal adhesions were positive for clathrin. In addition, as shown in Figure 9B, a previously published technique for assessing colocalisation by analysing the effect on Pearson's correlation coefficient of shifting one channel relative to another one pixel at a time confirms the lack of colocalisation of clathrin and focal adhesions (39,42). Finally, we performed two colour live-cell TIRF imaging to specifically determine if clathrin was transiently recruited to disassembling focal adhesions and as depicted in Figure 10C, this was not observed.

We chose to use a fluorescent tagged integrin (in this case GFP-β3-integrin) as our focal adhesion marker as it represents a potential endocytic cargo localised to the sites of focal adhesions, and therefore has the potential to colocalise with clathrin during endocytosis. However β3-integrin might not be present in all focal adhesions, thus we performed similar analyses with paxillin-mRFP. Importantly in control studies we assessed the colocalisation of GFP-β3-integrin and paxillin-mRFP. As can be seen in Figure S4, all regions of paxillin-mRFP also contain GFP-β3-integrin.

As with GFP-β3-integrin, no colocalisation was observed between paxillin-mRFP and clathrin-GFP in either static images of migrating cells or by analysing whether clathrin was recruited to regions of disassembling focal adhesions, as in the previous analysis (Figure S5A). Thus, these results confirm that clathrin-mediated endocytosis does not contribute directly to focal adhesion disassembly in this system. Similarly, although siRNA silencing of caveolin1 did not inhibit EGF-directed chemotactic invasion of MDA-MB-231 cells we also verified that caveolin1 also did not colocalise with GFP-β3-integrin (Figure S5B).

**Figure 11:**
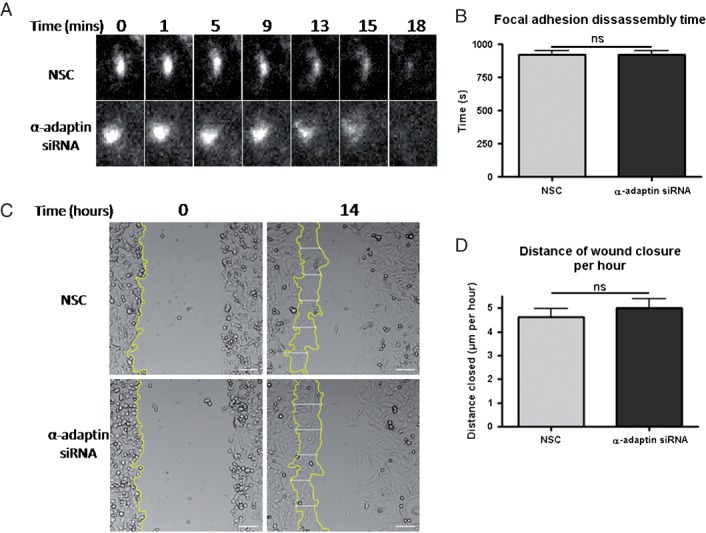
siRNA silencing of α-adaptin does not inhibit focal adhesion disassembly during wound healing. A) Disappearance of paxillin-mRFP over time using NSC and α-adaptin siRNA. B) Graph showing mean focal adhesion disassembly time for cells treated with NSC and α-adaptin siRNA. *n* ≥ 100 focal adhesions from 20 cells per treatment. C) Wound healing assay with α-adaptin knock-down. Representative images of wounds at time 0 and after 14 h. Lines denote edge of wound at both time points. Distances between cells at time 0 and 14 h were measured at various points in each image to give an average distance migrated in NSC and α-adaptin siRNA treated cells. D) Quantification of distance moved by siRNA treated cells. *n* = 22 fields of view per treatment.

As a final means to investigate a potential role for clathrin-mediated endocytosis in focal adhesion disassembly we assessed whether inhibition of clathrin-mediated endocytosis with siRNA against α-adaptin altered the rate of focal adhesion disassembly measured by timelapse TIRF microscopy of mRFP-paxillin, a previously employed assay strategy (7). However, as cells treated with α-adaptin siRNA do not readily migrate into EGF-containing agarose spots these experiments were performed with MDA-MB-231 cells migrating in a wound healing assay. Consistent with our colocalisation analysis (Figure 9), we found no differences in the time taken for focal adhesions to disassemble in knock-down cells compared to control (Figure 11A and B), indicating that clathrin is not required for disassembly of focal adhesions in migrating cells. Furthermore, as α-adaptin siRNA did not reduce the rate of wound healing (Figure 11C and D) it may be that EGFR trafficking is the main role of clathrin-mediated endocytosis during chemotactic invasion and during wound healing this pathway is not required.

## Discussion

The role of endocytosis in chemotactic invasion has not been adequately investigated. It has been suggested in some models that endocytosis is involved in focal adhesion disassembly (10,11,16,17). Furthermore, endocytosis can play a role in the modulation of receptor signalling (5,43–45). Therefore we conducted a series of studies to determine the role(s) of endocytosis in driving chemotactic invasion of breast cancer-derived cells. Increased understanding of how endocytosis regulates chemotactic invasion may assist in developing therapeutic interventions that might target tumour cell invasion and metastasis.

We firstly sought to establish whether endocytosis was important for chemotactic invasion of MDA-MB-231 cells towards EGF. Using Dynasore to inhibit dynamin-dependent endocytosis we found that far fewer cells were able to migrate, and that the migration of those which could still migrate was significantly impaired (Figure 1). In addition, we obtained identical results using an unrelated pancreatic cancer-derived cell line, further emphasising the reproducibility of the assay used and the necessity for dynamin-dependent endocytosis in chemotactic invasion (Figure 2). Because Dynasore had such a profound effect on migration we next investigated whether this might be as a result of a role involving endocytosis of receptors (noteably EGFR) or focal adhesion components (e.g. integrins). We found that Dynasore caused an accumulation of EGFR towards the front of migrating cells, however did not affect the localisation of focal adhesion components (Figures 3 and 4).

Because multiple endocytosis pathways are dynamin-dependent (46–48) we employed an RNAi approach to inhibit specific routes of endocytosis and further narrow down the role of endocytosis in chemotactic invasion. Upon inhibition of clathrin-mediated endocytosis by siRNA against α-adaptin we again saw a significant reduction in the number of cells able to migrate, demonstrating this to be a pathway necessary for migration towards EGF (Figure 5). Conversely, using siRNA against caveolin1 to inhibit caveolar endocytosis, we saw no alteration in cell migration.

Aspects of these results are in contrast to those reported by other groups. In 2012 Urra et al. inhibited caveolin1 in MDA-MB-231 cells and found this inhibition to decrease migration of cells in a wound healing model (49). The authors therefore suggested a role for caveolin1 in enhancing the metastatic potential of cancer cells. However, another study found that expression of caveolin1 led to an inhibition of chemotaxis and chemoinvasion in melanoma cells and suggested a role for the protein in inhibiting cell migration (50). Thus controversy remains, with different groups pointing to a role for caveolin1 in either promoting or inhibiting migration in some way (49–53). In order to verify our results we also undertook TIRF imaging of caveolin1-mRFP in conjunction with GFP-β3-integrin in cells migrating towards EGF and found no notable colocalisation between the two (Figure S5B). We therefore conclude that caveolar endocytosis is unnecessary for EGF-directed chemotactic invasion of MDA-MB-231 cells. However, it may be necessary for other modes of migration.

As clathrin-mediated endocytosis was required for chemotactic invasion towards EGF we tested for colocalisation between clathrin and EGFR and found a high degree of colocalisation between the two (Figure 6). When we tested colocalisation of EGFR with caveolin1 we found that this was negligible compared to the colocalisation with clathrin. This indicates a clathrin-dependent internalisation route for the EGFR in cells migrating in an EGF-dependent manner. In the literature there is much support for a clathrin-dependent route of EGFR endocytosis (20,21,54,55) but also contrary evidence supporting a clathrin-independent route in certain situations (22,23,56,57). In support of our findings, previous work by Huang et al. and Rappoport and Simon demonstrated that in non-migrating cells the EGFR enters primarily through clathrin-coated pits and not through caveolae (20,21). In addition to assessing colocalisation of clathrin and EGFR we were able to image instances of both simultaneously disappearing from the TIRF field (Figure 7A and B). During this live-cell imaging we also saw instances of EGFR clusters devoid of clathrin moving laterally adjacent to the plasma membrane, likely to be post-endocytic structures or post-Golgi vesicles (Figure 7C). This observation suggests that some EGFR in our analysis in Figure 6 may be EGFR of this category and therefore the colocalisation of EGFR clusters with clathrin-coated pits/vesicles on the plasma membrane could be even higher than originally thought.

An emerging idea is that polarised vesicle trafficking is involved in the regulation of directed cell migration (6). A link between polarised trafficking of cell adhesion components has been shown in a number of cell types (11,58–60). The occurrence of polarised trafficking of chemoattractant receptors is less well studied than that of focal adhesions, but may play a role in regulation of directed cell migration (61). A recent study by Assaker et al. found that particular endocytic proteins were necessary to control the trafficking, and hence spatial polarisation, of receptor tyrosine kinases during Drosophila boarder cell migration (62). Another study by Belleudi et al. found endocytosis and polarised recycling of the keratinocyte growth factor receptor regulated receptor polarisation at the plasma membrane, and that this was necessary for keratinocyte migration (63). Upon inhibition of dynamin-dependent endocytosis we have already shown a redistribution of EGFR localisation towards the front of migrating cells (Figure 3). This implies that EGFR internalisation is polarised to the front of migrating cells, as inhibition of endocytosis caused a build up endocytosis cargo in this region. This led us to test whether clathrin-mediated endocytosis is polarised in MDA-MB-231 cells during EGF-directed chemotactic invasion. As expected, we saw an increased number of internalisation events at the front of cells migrating towards EGF rather than at the middle or back (Figure 8). In addition to the necessity for clathrin-mediated endocytosis for migration in this model, these results suggest that polarised endocytosis of the EGFR is important for migration in this breast cancer cell line.

There has been data reported suggesting a role for clathrin-mediated endocytosis of integrins from disassembling focal adhesions (34,35). However, in other studies this has not been observed (7,36). As inhibition of endocytosis significantly decreased chemotactic invasion, we tested whether there is a role for clathrin-mediated endocytosis in focal adhesion disassembly. However, we found no significant colocalisation between clathrin and the focal adhesion markers β3-integrin and paxillin, including specifically during focal adhesion disassembly (Figure 10 and Figure S5A). In addition to chemotactic invasion studies we also undertook wound healing studies using MDA-MB-231 cells where we found knock-down of α-adaptin had no effect on focal adhesion disassembly time. Wound healing was assessed here because silencing of α-adaptin had such a profound effect on chemotactic invasion that we could not perform these analyses in that model.

Our data in MDA-MB-231 wound healing corroborates with wound healing results from other studies, including those from Fletcher et al. in MDCK cells where they also found no role for endocytosis during focal adhesion disassembly (7). The contrast in these results compared to some studies performed in fibroblasts indicates that effects seen on focal adhesions may be cell type and/or migratory stimulus specific and that while there may be a requirement for clathrin in focal adhesion disassembly in fibroblasts, this is not the case during chemotactic invasion of MDA-MB-231 breast cancer cells (34,35).

In addition we found that knocking down α-adaptin had no effect on the wound closure rate of MDA-MB-231. This indicated that while some pathways may be necessary for one process they may be dispensable for other, otherwise similar, phenotypes. Furthermore, this suggests that EGFR trafficking might be a critical role of clathrin-mediated endocytosis during chemotactic invasion towards EGF in MDA-MB-231 cells, but not necessary for wound healing.

Taken together these results suggest a model where dynamin and clathrin are necessary for chemotactic invasion in MDA-MB-231 cells, but caveolin1 is not. We also establish that clathrin-mediated endocytosis is not necessary for focal adhesion disassembly in this cell line. We propose that the main route of EGFR internalisation is clathrin-mediated endocytosis, which is polarised towards the front of migrating cells. Comparison of these results with other studies provides significant evidence for differences between cell types, as well as migratory models. Thus, there results suggest that a more systematic and empirical approach is required before broad conclusions can be suggested to describe the role(s) of endocytosis in directed cell motility.

## Materials and methods

### Cell culture

MDA-MB-231 (ATCC), PDAC and PDACs cells (gift from Kurt Anderson, Beatson Institute) were incubated at 37°C in 5% (v/v) CO_2_. Cells were maintained in Dulbecco modified Eagle medium (DMEM, Lonza) supplemented with 10% foetal calf serum (FCS) (Gibco) and 1% Pen-Strep (Gibco). PDACs cells are stably transfected versions of PDAC cells with a Src FLIM/FRET probe and, for the purpose of this manuscript, are referred to as PDACs (rather than PDAC) to denote the difference from regular PDAC cells.

### Transfection and plasmid constructs

cDNA transfections of MDA-MB-231 cells were achieved using Lipofectamine 2000 (Life Technologies). Cells were plated to approximately 80% confluency 24 h prior to transfection. Assays with these cells were performed 24 or 48 h post transfection (depending on the cDNA construct and assay being performed).

cDNA transfection of PDAC cells was achieved using electroporation. Cells were plated 24 h prior to transfection and on the day of transfection were trypsinised and counted. Per transfection we used 2.5 × 10^5^ cells with 4 µg cDNA, 100 μL electroporation solution (Ingenio) and programme X-01 on the Lonza Nucleofector 2b device.

The construct encoding clathrin-dsRed was a gift from Thomas Kirchhausen (Harvard Medical School), GFP-β3-integrin was a gift from Jonathan Jones (Northwestern University Medical School), paxillin-mRFP was a gift from Maddy Parsons (King's Collage London), Cav1–mRFP was a gift from Ari Helenius (Swiss Federal Institute of Technology Zurich) and EGFR-GFP was a gift from Alexander Sorkin (University of Pittsburgh).

siRNA transfections were achieved using DharmaFECT 1 (Thermo Scientific). siRNA constructs were obtained from Thermo Scientific. Cells were plated to approximately 40% confluency 24 h prior to transfection. Assays with these cells were performed 72 h post transfection.

### Endocytosis assays

Uptake assays were used to assess the effect of Dynasore treatment, α-adaptin knock-down and caveolin1 knock-down on endocytosis. 24 h prior to the assay cells were plated onto sterilised glass cover slips. For assays with Dynasore each well was rinsed with serum free media (SFM) before incubation with SFM containing 80 μM Dynasore (Sigma), or the equivalent volume of dimethyl sulphoxide (DMSO), for 30 min. A 10 min incubation with just SFM was used for assays utilising siRNA transfected cells. After this initial incubation cells were then further incubated for 5 min with either transferrin (Alexa Fluor 568 conjugate, 10 µg/mL, Life Technologies) or cholera toxin B subunit (CTxB, Alexa Fluor 555 conjugate, 1 µg/mL, Life Technologies) diluted in SFM. After incubation cells incubated with transferrin were washed with acid PBS twice followed by a PBS wash; cells incubated with CTxB were washed twice with SFM followed by a PBS wash. Cover slips were fixed in 4% paraformaldehyde (PFA, Electron Microscopy Sciences) for 5 min. Cover slips were fixed to slides using ProLong Gold (Life Technologies) and imaged using a Nikon TE300 Inverted Epi-fluorescence microscope with a 60× oil objective lens. Random fields of view from each slide were recorded and the light intensity of 20 random cells per slide was analysed.

### Transwell assays

Chemotaxis assays were carried out using Transwell plates (24 mm diameter polycarbonate inserts with 8 µm pores, Corning Life Sciences). EGF was used as the chemoattractant at a concentration of 1 µg/mL EGF. The Dynasore group was treated with 80 μM, and 5 × 10^5^ cells were plated into each insert and Transwells were incubated at 37°C, 5% CO_2_ for 5 h. After incubation, inserts were washed in PBS and fixed in 4% PFA for 5 min. After fixing, membranes were treated with Vectashield mounting medium with DAPI (4′,6-diamidino-2-phenylindole) (Vector Laboratories) to stain cell nuclei and fixed to slides. Imaging was done using a Nikon TE300 Inverted Epi-fluorescence microscope with a 40× oil objective lens. Random fields of view from each slide were recorded and numbers of migrated cells in each field were counted.

## Western blot

Cells were lysed in 1% Triton X-100 (Sigma) in PBS with protease inhibitors (complete mini EDTA-free protease inhibitor cocktail, Roche). Proteins were separated by SDS–PAGE and transferred from the gel onto Immobilon-FL membrane (Millipore). The membrane was blotted with primary antibody overnight at 4°C and then with secondary antibody for 2 h at room temperature. Primary antibodies used were polyclonal rabbit anti-α-adaptin (Life Technologies), polyclonal rabbit anti-caveolin1 (BD Biosciences) and monoclonal mouse anti-tubulin (Sigma). Secondary antibodies used were anti-rabbit IRDye 800 and anti-mouse 680 (Li-cor) for use in Odyssey. Quantification of knock-downs was performed using Odyssey relative to control tubulin.

### Agarose spot assay

A recently developed agarose spot assay was utilised to investigate chemotactic invasion as described in Wiggins and Rappoport (24). For TIRF imaging or overnight timelapses cell imaging media [10 mM Hepes (Sigma), 9.7 g Hank's Balanced Salt Solution (Life Technologies) in 1 L dH_2_O, pH 7.4] with low serum content (0.1% FCS) was used. For the Dynasore treatment assays the low serum media contained Dynasore at 80 μM and the control group was given the same volume of DMSO (Sigma).

### Imaging and image analysis

Overnight timelapses (for agarose spot assays) were performed on a Nikon eclipse Ti inverted microscope using a 10× air objective lens (Nikon Instruments). TIRF imaging was carried out using a Nikon A1-R Ti TIRF system with a 60× oil objective lens (CFI TIRF Apo 60× oil NA 1.49, Nikon) (Nikon Instruments).

For quantification of fluorescence for the endocytosis assays imagej was used. For all other image analysis NIS elements (version 3.1) was used. For cell tracking analysis the manual tracking software in NIS elements was used.

For colocalisation analysis of clathrin-dsRed or caveolin1-mRFP with EGFR-GFP 50 clusters of EGFR in the green channel were circled, the number of these corresponding to a cluster of clathrin/caveolin1 in the red channel was then recorded. To assess colocalisation by chance, the same 50 circled regions were moved slightly so that they corresponded to regions where EGFR-GFP was not present; the number of these control regions which corresponded to a clathrin/caveolin1 cluster was recorded as the control value. For assessing colocalisation of clathrin with β3-integrin or paxillin-mRFP a 10 µm line was drawn across two disassembling focal adhesions and a kymograph across this region over 40 min was made.

For analysis involving splitting the cell into front, middle and back regions of migrating cells control timelapses (i.e. no inhibition of migration) were used to establish the overall direction taken by cells within agarose spots. We calculated the angle of cells from the last point on the timelapses with respect to a line perpendicular to the edge of the agarose spot. From this analysis it was found that on average cells migrate to within 2.80° (±2.3°) to the perpendicular, effectively meaning that the line perpendicular to the agarose spot edge can be used to estimate the direction of migration. For analysis involving establishing the front, middle and back of migrating cells we therefore used a line perpendicular to the edge of the agarose spot and divided this into three equal lengths. All analyses were normalised to the area of each region to account for their different sizes (unless stated otherwise).

### Wound healing assay

For focal adhesion disassembly experiments, cells transfected with α-adaptin siRNA and paxillin-mRFP were plated onto 35-mm glass-bottomed dishes (MatTek Corporation) 24 h prior to wounding to give a confluent layer of cells. Cells were wounded using a 200 μL pipette tip. Cells at the edge of the wound were imaged using a Nikon A1-R Ti TIRF system with a 60× oil objective lens.

For experiments on the effect of α-adaptin knock-down on wound closure rate, cells were plated onto 35-mm glass-bottomed dishes and transfected with siRNA 72 h prior to wounding with a 200 μL pipette tip. Wounds were imaged every 15 min for 14 h with a Nikon eclipse Ti inverted microscope using a 10× air objective lens. Images were analysed using NIS elements (version 3.1) and average distance of migration was recorded.

## Supporting Information

Figure S1 **Endocytosis controls for Dynasore treatment.** A) Representative images of transferrin uptake (left panels) and cholera toxin B subunit uptake (right panels) with DMSO (as control) or Dynasore. B) Quantification of inhibition of transferrin and cholera toxin B subunit uptake by Dynasore treatment. Dynasore inhibited transferrin uptake by 60% and inhibited cholera toxin uptake by 52%. *n* = 60 cells per treatment.

Figure S2 **Imaging of clathrin-dsRed and EGFR-GFP in cells with no EGF stimulation.** Scale bars are 10 µm.

Figure S3 **Controls for α-adaptin and caveolin1 siRNA.** A) Western blot analysis of α-adaptin and caveolin1 knock-down in siRNA treated cells. B) Representative images and quantification of transferrin uptake and cholera toxin B subunit uptake inhibition in siRNA treated cells. Inhibition of transferrin uptake in α-adaptin siRNA treated cells (45%) and inhibition of cholera toxin B subunit uptake in caveolin1 siRNA treated cells (50%). *n* = 3 for knock-down analysis, *n* = 60 cells per treatment for transferring and cholera toxin uptake assays.

Figure S4 **Colocalisation analysis of focal adhesion markers in migrating cells.** Representative image showing a high degree of colocalisation between paxillin-mRFP and GFP-β3-integrin.

Figure S5 **Lack of colocalisation between endocytosis markers and focal adhesions.** A) Representative images of paxillin-mRFP and clathrin-GFP in migrating cells. Scale bars are 10 µm. Kymograph to show lack of colocalisation between paxillin-mRFP labelled focal adhesions and clathrin-GFP over time. A 10 µm line was drawn across two disassembling focal adhesions and a kymograph across this region demonstrated no colocalisation of clathrin during focal adhesion disassembly. B) Representative image showing a lack of colocalisation between caveolin1-mRFP and GFP-β3-integrin. Scale bars are 10 µm. Arrows show direction of migration.
