# Supplementary material for: Polarised Clathrin-Mediated Endocytosis of EGFR During Chemotactic Invasion
Source: Traffic. 2014 Mar 20;15(6):648–64. doi: 10.1111/tra.12165 (PMC4309520; doi:10.1111/tra.12165)
Supplement: Figure S1 — Endocytosis controls for Dynasore treatment. A) Representative images of transferrin uptake (left panels) and cholera toxin B subunit uptake (right panels) with DMSO (as control) or Dynasore. B) Quantification of inhibition of transferrin and cholera toxin B subunit uptake by Dynasore treatment. Dynasore inhibited transferrin uptake by 60% and inhibited cholera toxin uptake by 52%. n = 60 cells per treatment. [file tra0015-0648-sd1.pdf]

Figure S1

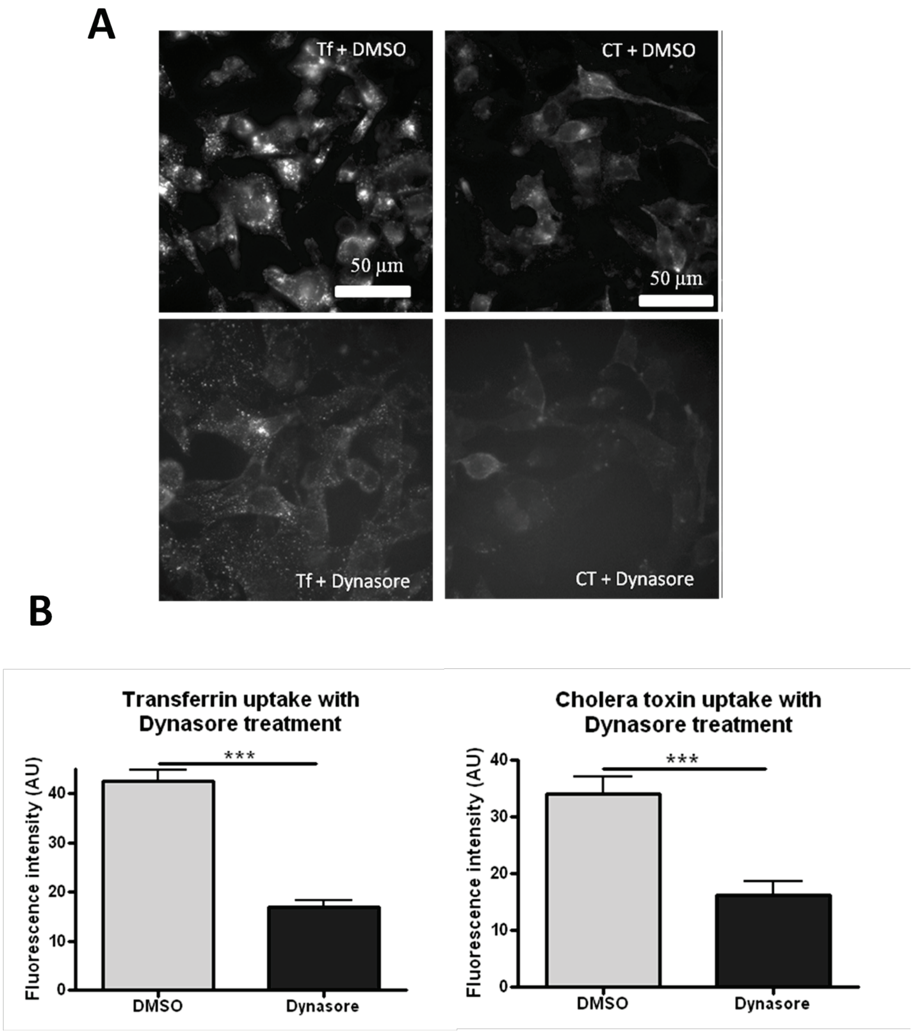

Figure S1. Endocytosis controls for Dynasore treatment. A) Representative images of transferrin uptake (left panels) and cholera toxin B subunit uptake (right panels) with DMSO (as control) or Dynasore. B) Quantification of inhibition of transferrin and cholera toxin B subunit uptake by Dynasore treatment. Dynasore inhibited transferrin uptake by 60% and inhibited cholera toxin uptake by 52%. n = 60 cells per treatment.

Figure S2

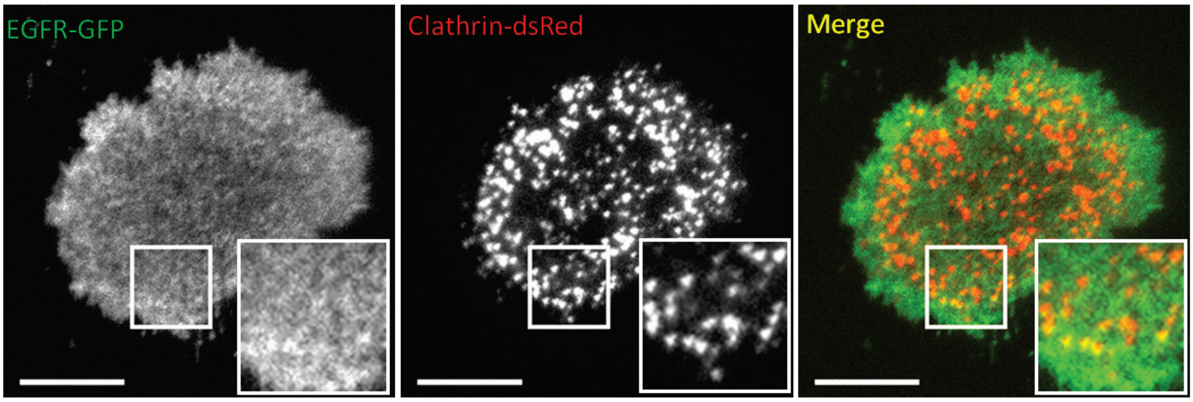

Figure S2. Imaging of clathrin-dsRed and EGFR-GFP in cells with no EGF stimulation. Scale bars are 10 μm.

Figure S3

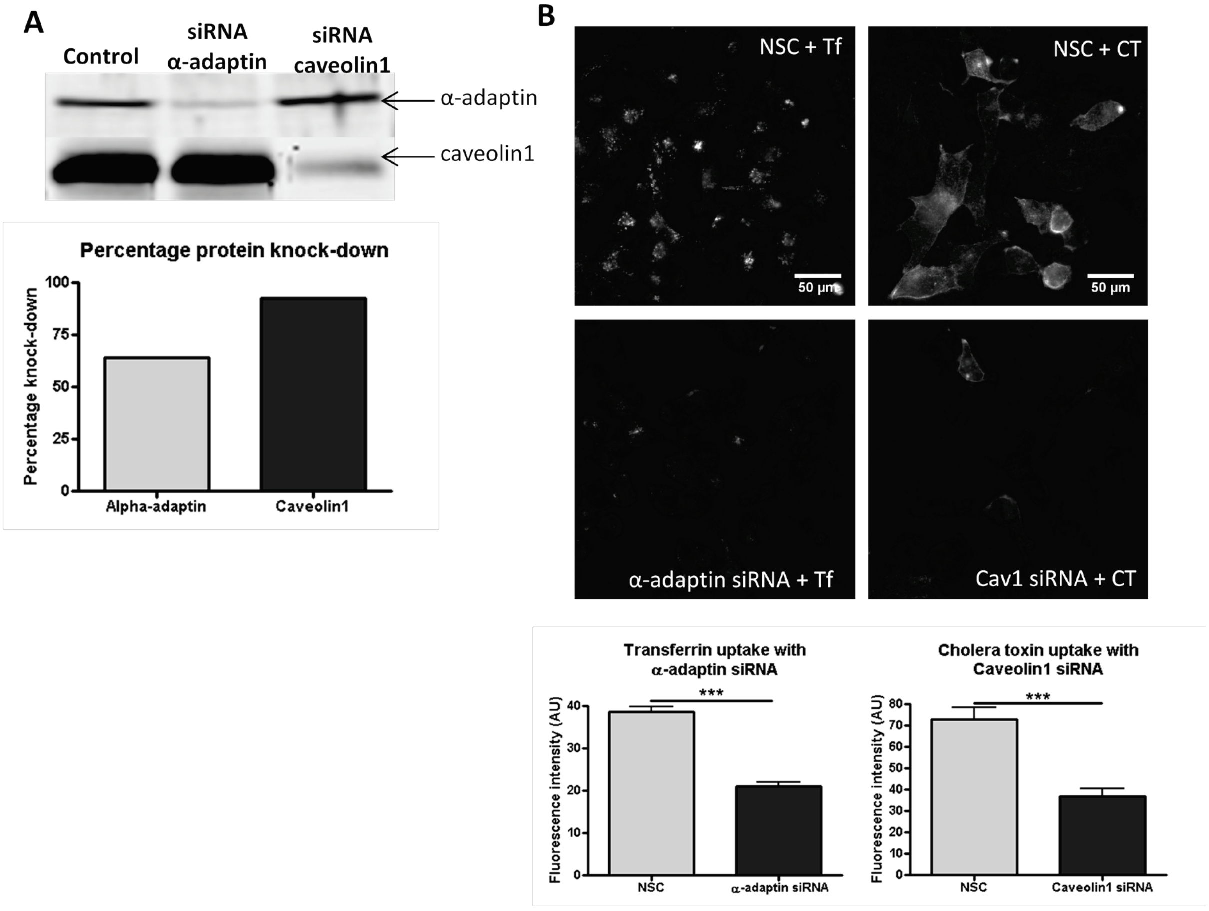

Figure S3. Controls for α-adaptin and caveolin1 siRNA. A) Western blot analysis of α-adaptin and caveolin1 knock-down in siRNA treated cells. B) Representative images and quantification of transferrin uptake and cholera toxin B subunit uptake inhibition in siRNA treated cells. Inhibition of transferrin uptake in α-adaptin siRNA treated cells (45%) and inhibition of cholera toxin B subunit uptake in caveolin1 siRNA treated cells (50%). n = 3 for knockdown analysis, n = 60 cells per treatment for transferring and cholera toxin uptake assays.

Figure S4

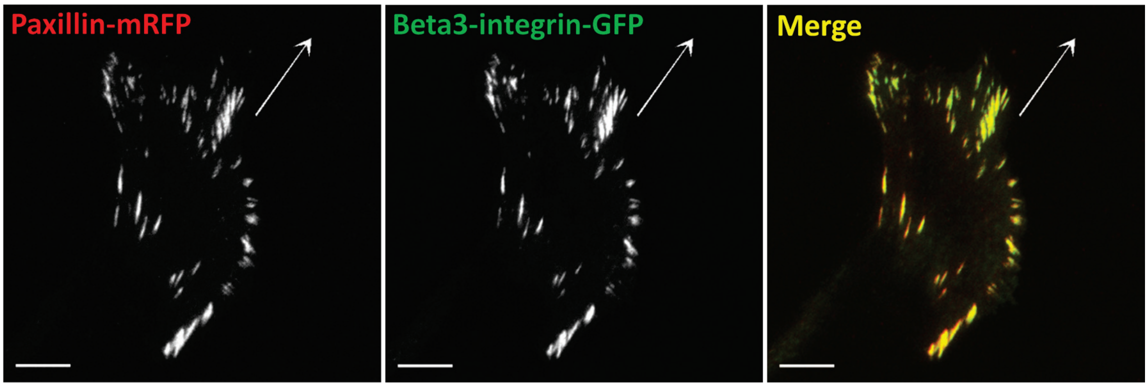

Figure S4. Colocalisation analysis of focal adhesion markers in migrating cells. Representative image showing a high degree of colocalisation between paxillin-mRFP and GFP-β3-integrin.

Figure S5

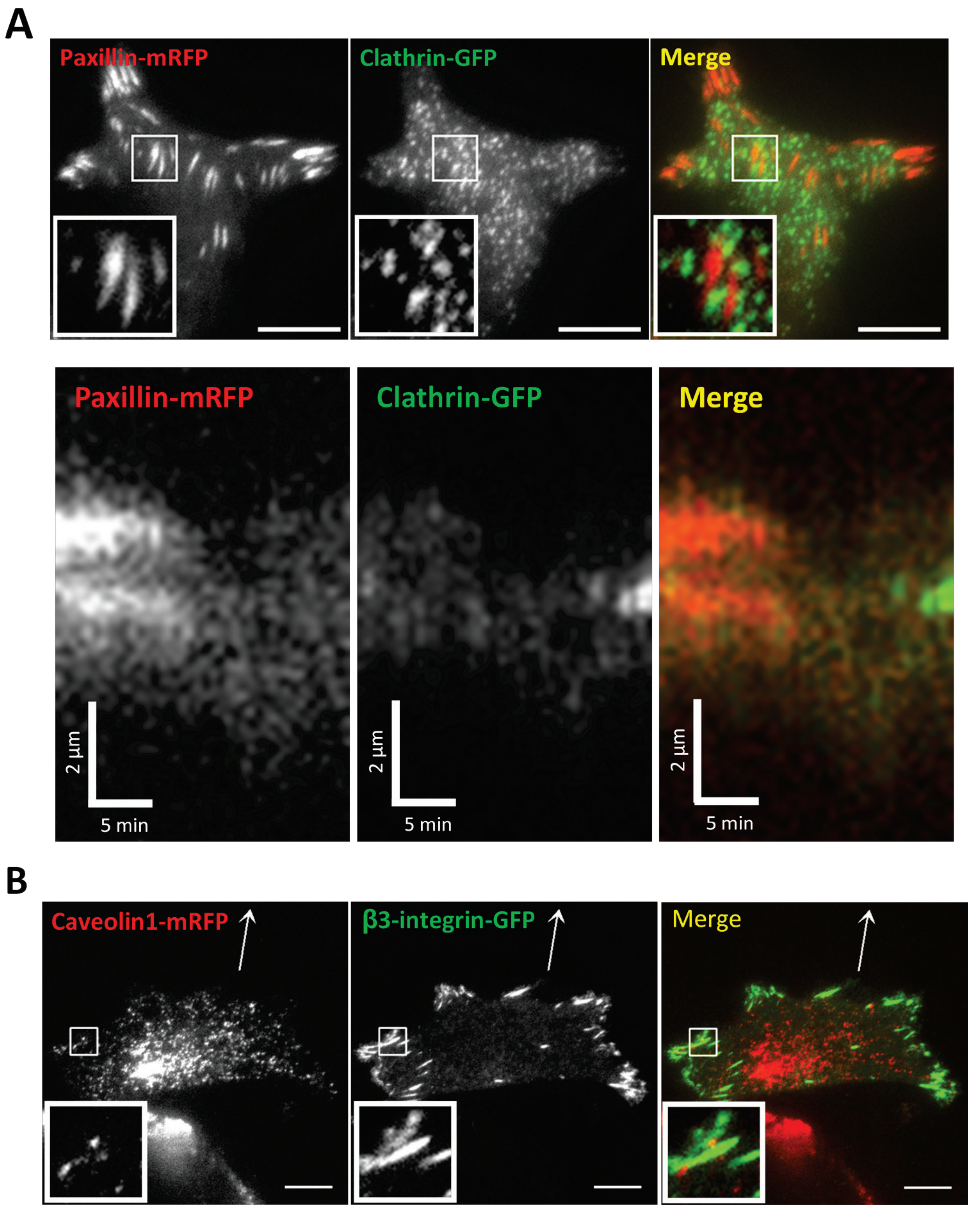

Figure S5. Lack of colocalisation between endocytosis markers and focal adhesions. A) Representative images of paxillin-mRFP and clathrin-GFP in migrating cells. Scale bars are 10 µm. Kymograph to show lack of colocalisation between paxillin-mRFP labelled focal adhesions and clathrin-GFP over time. A 10 µm line was drawn across two disassembling focal adhesions and a kymograph across this region demonstrated no colocalisation of clathrin during focal adhesion disassembly. B) Representative image showing a lack of colocalisation between caveolin1-mRFP and GFP-β3-integrin. Scale bars are 10 µm. Arrows show direction of migration.
